# Gender differences in stage at diagnosis and preoperative radiotherapy in patients with rectal cancer

**DOI:** 10.1186/s12885-020-07195-4

**Published:** 2020-08-14

**Authors:** Cristina Sarasqueta, Mª Victoria Zunzunegui, José María Enríquez Navascues, Arrate Querejeta, Carlos Placer, Amaia Perales, Nerea Gonzalez, Urko Aguirre, Marisa Baré, Antonio Escobar, José María Quintana, on behalf of the REDISSEC-CARESS/CCR Group, Jose María Quintana López, Marisa Baré Mañas, Maximino Redondo Bautista, Eduardo Briones Pérez de la Blanca, Nerea Fernández de Larrea Baz, Cristina Sarasqueta Eizaguirre, Antonio Escobar Martínez, Francisco Rivas Ruiz, Maria M. Morales-Suárez-Varela, Juan Antonio Blasco Amaro, Isabel del Cura González, Inmaculada Arostegui Madariaga, Amaia Bilbao González, Nerea González Hernández, Susana García-Gutiérrez, Iratxe Lafuente Guerrero, Urko Aguirre Larracoechea, Miren Orive Calzada, Josune Martin Corral, Ane Antón-Ladislao, Núria Torà, Marina Pont, María Purificación Martínez del Prado, Alberto Loizate Totorikaguena, Ignacio Zabalza Estévez, José Errasti Alustiza, Antonio Z. Gimeno García, Santiago Lázaro Aramburu, Mercè Comas Serrano, Jose María Enríquez Navascues, Carlos Placer Galán, Amaia Perales Antón, Iñaki Urkidi Valmaña, Jose María Erro Azkárate, Enrique Cormenzana Lizarribar, Adelaida Lacasta Muñoa, Pep Piera Pibernat, Elena Campano Cuevas, Ana Isabel Sotelo Gómez, Segundo Gómez-Abril, F. Medina-Cano, Julia Alcaide, Arturo Del Rey-Moreno, Manuel Jesús Alcántara, Rafael Campo, Alex Casalots, Carles Pericay, Maria José Gil, Miquel Pera, Pablo Collera, Josep Alfons Espinàs, Mercedes Martínez, Mireia Espallargues, Caridad Almazán, Paula Dujovne Lindenbaum, José María Fernández-Cebrián, Rocío Anula Fernández, Julio Mayol Martínez, Ramón Cantero Cid, Héctor Guadalajara Labajo, María Alexandra Heras Garceau, Damián García Olmo, Mariel Morey Montalvo

**Affiliations:** 1 Biodonostia Health Research Institute - Donostia University Hospital, Paseo Dr. Beguiristain s/n (Gipuzkoa), 20014 Donostia-San Sebastián, Spain; 2 Red de Investigación en Servicios de Salud en Enfermedades Crónicas (REDISSEC), Galdakao, Bizkaia Spain; 3 Professeure honoraire. École de santé publique (ESPUM) Departement de médecine sociale et préventive, Université de Montréal, Pavillon 7101, salle 3111 7101, Avenue du Parc Montréal, Québec, H3N 1X9 Canada; 4 Department of General and Digestive Surgery, Donostia University Hospital, Paseo Dr. Beguiristain 109 (Gipuzkoa), 20014 Donostia-San Sebastián, Spain; 5 Radiotherapic Oncology, Donostia University Hospital, Paseo Dr. Beguiristain 109 (Gipuzkoa), 20014 Donostia-San Sebastián, Spain; 6 Biodonostia Health Research Institute, Paseo Dr. Beguiristain s/n (Gipuzkoa), 20014 Donostia-San Sebastián, Spain; 7 Clinical Epidemiology and Cancer Screening, Corporació Sanitaria Parc Taulí, Parc Taulí 1, 08208 Sabadell, Barcelona, Spain; 8 Research Unit, Hospital Basurto, Avda Montevideo, 18, 48013 Bilbao, Bizkaia Spain; 9 Research Unit, Galdakao-Usansolo Hospital, Labeaga Auzoa, 48960 Galdakao, Bizkaia Spain

**Keywords:** Rectal Cancer, Gender, Tumor staging, Adjuvant radiotherapy, Delayed diagnosis

## Abstract

**Background:**

Few studies have examined gender differences in the clinical management of rectal cancer. We examine differences in stage at diagnosis and preoperative radiotherapy in rectal cancer patients.

**Methods:**

A prospective cohort study was conducted in 22 hospitals in Spain including 770 patients undergoing surgery for rectal cancer. Study outcomes were disseminated disease at diagnosis and receiving preoperative radiotherapy. Age, comorbidity, referral from a screening program, diagnostic delay, distance from the anal verge, and tumor depth were considered as factors that might explain gender differences in these outcomes.

**Results:**

Women were more likely to be diagnosed with disseminated disease among those referred from screening (odds ratio, confidence interval 95% (OR, CI = 7.2, 0.9–55.8) and among those with a diagnostic delay greater than 3 months (OR, CI = 5.1, 1.2–21.6). Women were less likely to receive preoperative radiotherapy if they were younger than 65 years of age (OR, CI = 0.6, 0.3–1.0) and if their tumors were cT3 or cT4 (OR, CI = 0.5, 0.4–0.7).

**Conclusions:**

The gender-specific sensitivity of rectal cancer screening tests, gender differences in referrals and clinical reasons for not prescribing preoperative radiotherapy in women should be further examined. If these gender differences are not clinically justifiable, their elimination might enhance survival.

## Background

Some studies have reported that women have more advanced stages at diagnosis in colorectal cancer [1–6]. In rectal cancer, the evidence is scarce and inconclusive [7–9].

Gender differences in the percentage of patients who have disseminated disease at diagnosis of rectal cancer could be due to differences in diagnostic delay. In a systematic review of 54 studies on pre-hospital delay in the diagnosis of colorectal cancer [10], 5 studies examined gender differences and no robust conclusions could be drawn concerning gender differences in diagnostic delay [11–15]. More recent studies [7, 16] have reported longer intervals from first clinical symptoms to diagnosis in women than in men. To date, none of the studies comparing diagnostic delay in men and women with rectal cancer have attempted to explain the gender differences observed.

Five studies have reported that women are less likely to receive preoperative radiotherapy [17–21], but how a patient’s gender influences clinical management of rectal cancer is seldom examined. The aforementioned findings prompt us to question under which circumstances the clinical management of rectal cancer vary between men and women.

The aim of this study was to investigate potential differences in stage at diagnosis and use of preoperative radiotherapy between men and women with rectal cancer. Specifically, we sought to describe the magnitude of any gender differences, and to examine whether they vary by age, or clinical or tumor characteristics.

## Methods

### Study design and participants

The CARESS colorectal cancer health services research study was a prospective cohort study conducted at 22 hospitals in 5 autonomous regions in Spain. Patients were eligible if they had undergone curative or palliative surgery for a first-time diagnosis of colorectal cancer made in one of the participating hospitals between April 2010 and December 2012. All cancers were histologically verified carcinomas of the colon or rectum. In the study period, 2986 eligible patients were recruited and of these 237 declined the invitation to participate (7.9% of the men and 8.1% of the women). The remaining 2749 patients were included, 1979 with colon and 770 with rectal cancer. All hospital Institutional Review Boards approved the study and all participants signed an informed consent form. For this sub-analysis, we have included the 770 patients with rectal cancer, 522 men and 248 women [22, 23].

The STROBE reporting guidelines for cross-sectional studies were followed [24].

### Variables

Data were obtained from medical records at cohort inception [22].

The outcomes of interest are: 1) disseminated or stage IV disease, assessed according to the Pathologic staging of the 7th Edition of the American Joint Committee on Cancer (AJCC) TNM Cancer Staging Manual, considering data on loco-regional staging obtained from histopathological reports, and the diagnosis of distant metastasis based on chest radiography and liver ultrasonography or tomography; and 2) use of preoperative radiotherapy (yes vs no), reflecting whether or not the patient received radiotherapy before surgery.

We refer to gender as a concept encompassing the entangled social and biological differences between men and women. Gender was the explanatory variable in the statistical analyses, 1 for women and 0 for men.

Additionally, we considered the following factors which, according to the literature, might confound or modify the associations between gender and the outcomes of interest:

Patient characteristics: a) age: categorized into three groups (< 65, 65 to 80, > 80 years), employing the categorization used in previous articles based on this cohort [23]; b) screening status: patients being categorized as screen detected (if diagnosed after a positive fecal occult blood [FOB] test performed as part of population or opportunistic screening) or symptomatic (if diagnosed after having sought medical attention through primary care or emergency services); c) comorbidities: measured using the Charlson comorbidity index [25], classified into three groups (0, 1, and 2 or more); and d) diagnostic delay: number of days between the first contact with a physician and the first positive histological diagnosis, categorized into ≤ and > than 3 months; in patients referred from screening, the date of signing the FOB test report was considered the first contact.

Tumor characteristics: a) distance from the anal verge (< 5, 5–10, > 10 cm), based on endoscopic and magnetic resonance imaging (MRI) findings; b) pretreatment tumor depth, cT, coded according to the Clinical staging of the 7th edition of the AJCC TNM Cancer Staging Manual: the “T” indicates how deeply the primary tumor has grown into the bowel lining and was assessed by endorectal ultrasound, pelvic computed tomography and/or MRI; and c) histological type: grouped into adenocarcinoma, mucinous adenocarcinoma and undifferentiated carcinomas for the statistical analysis.

### Statistical analysis

For all the analysis, missing values were included in a separate category. Distributions of the outcomes and covariates were compared between men and women using Pearson’s chi-square test for nominal variables and chi-square test for trend for age. Gender-stratified analysis was conducted to examine the univariate association of each covariate with stage IV and with the use of preoperative radiotherapy, in men and women. For both outcomes, multiple logistic regression models were constructed using generalized estimating equations (GEEs), to account for clustering by hospitals, and fitted using an exchangeable correlation structure. To test for a modifying effect of gender, multiplicative interactions between gender and each covariate were assessed. Such interactions were considered significant if the *p*-value obtained in the likelihood ratio (LR) test was ≤0.05.

For each significant interaction, analyses were performed stratifying by the values of the variable interacting with gender. The odds ratios for women versus men were estimated using the 95% confidence intervals of the point estimates in the GEE logistic regression models and adjusting for age, comorbidity and any tumor characteristics associated with each outcome with *p* < 0.20 in the previous gender-stratified bivariate analyses.

The statistical software used was IBM SPSS v 23. Power calculations for the logistic regression-based estimation of coefficients for binary variables and multiplicative interactions of binary variables were performed with the software developed by Demidenko [26], https://www.dartmouth.edu/~eugened/power-samplesize.php.

## Results

Table 1 shows that 10.5% of women and 6.5% of men had disseminated disease, with involvement of other organs, *p* = 0.09; while 50% of women received preoperative radiotherapy compared to 55.4% of men, *p* = 0.16. Men and women differed in level of comorbidity, men being more likely to have a Charlson index being ≥1 (*p* < 0.0005); in tumor histology, women being more likely to have an unknown histological type (*p* = 0.04); and in tumor depth, women being more likely to have cT4 disease (*p* < 0.0005). The interval between the first visit and diagnosis was longer in women, with delays > 3 months in 23.8% of women and just 15.7% of men (*p* = 0.02). No significant differences between men and women were found in age, percentage diagnosed through screening or tumor distance from the anal verge.

**Table 1:** Study outcomes and selected clinical characteristics by gender

	Men ***N*** = 522	Women ***N*** = 248	***p***-value^**1**^
Pathological stage			
0, I	192 (36.8)	100 (40.3)	0.09
II	144 (27.6)	53 (21.4)	
III	152 (29.1)	69 (27.8)	
IV	34 (6.5)	26 (10.5)	
Preoperative radiotherapy			
No	233 (44.6)	124 (50.0)	0.16
Yes	289 (55.4)	124 (50.0)	
Unknown	25 (4.8)	17 (6.9)	
Age			
65 years	221 (42.3)	108 (43.5)	0.68^2^
65–80 years	245 (46.9)	105 (42.3)	
> 80 years	56 (10.7)	35 (14.1)	
Charlson index			
0	258 (49.4)	165 (66.5)	< 0.0005
≥ 1	264 (50.6)	83 (33.5)	
Screening			
No	429 (82.2)	201 (81.0)	0.48
Yes	68 (13.0)	30 (12.1)	
Unknown	25 (4.8)	17 (6.9)	
Diagnostic delay			
≤ 3 months	374 (71.6)	155 (62.5)	0.02
> 3 months	83 (15.9)	59 (23.8)	
Unknown	65 (12.5)	34 (13.7)	
Distance from anal verge			
< 5 cm	100 (19.2)	57 (23.0)	0.16
5–10 cm	218 (41.8)	83 (33.5)	
> 10 cm	167 (32.0)	90 (36.3)	
Unknown	37 (7.1)	18 (7.3)	
Histological classification			
Adenocarcinoma	474 (90.8)	208 (83.9)	0.04
Mucinous adenocarcinoma	18 (3.4)	14 (5.6)	
Undifferentiated carcinomas	6 (1.1)	4 (1.6)	
Unknown	24 (4.6)	22 (8.9)	
Tumor depth (cT)			
cT1 - cT2	91 (17.4)	55 (22.2)	< 0.0005
cT3	273 (52.3)	120 (48.4)	
cT4	28 (5.4)	34 (13.7)	
Unknown	130 (24.9)	39 (15.7)	

Numbers in parentheses correspond to percentages

^1^ Pearson chi-square test

^2^ Chi-square test for trend

### Differences in disseminated disease

The gender-specific distribution of stage IV disease by categories of relevant clinical and tumor variables and gender interactions are reported in Table 2. Among women, stage IV disease was more common among screen-detected cases and those with an unknown referral route; while among men, a higher percentage of stage IV disease was observed when they had not been diagnosed through screening (*p* value for gender interaction = 0.002). Regarding diagnostic delay, among women, stage IV disease was more common in those with delay greater than 3 months, while among men, stage IV disease was more common in those with shorter delays (p value for gender interaction = 0.004).

**Table 2:** Association of stage IV disease with various factors in men and women

	Stage IV						
	Men			Women			***p***-value for gender interaction^**3**^
	N	n (%)	P^**1**^	N	n (%)	P^**1**^	
Age			0.38^2^			0.20^2^	0.80
< 65 years	221	17 (7.7)		108	14 (13.0)		
65–80 years	245	14 (5.7)		105	10 (9.5)		
> 80 years	56	3 (5.4)		35	2 (5.7)		
Charlson index			0.44			0.10	0.33
0	258	19 (7.4)		165	21 (12.7)		
≥ 1	264	15 (5.7)		83	5 (6.0)		
Screening			0.43			0.07	0.002
No	429	30 (7.0)		201	17 (8.5)		
Yes	68	2 (2.9)		30	5 (16.7)		
Unknown	25	2 (8.0)		17	4 (23.5)		
Diagnostic delay			0.16			0.37	0.004
≤ 3 months	374	29 (7.8)		155	13 (8.4)		
> 3 months	83	2 (2.4)		59	8 (13.6)		
Unknown	65	3 (4.6)		34	5 (14.7)		
Distance from anal verge			0.34			0.83	0.67
< 5 cm	100	3 (3.0)		57	5 (8.8)		
5–10 cm	218	15 (6.9)		83	10 (12.0)		
> 10 cm	167	12 (7.2)		90	10 (11.1)		
Unknown	37	4 (10.8)		18	1 (3.8)		
Histological classification			0.49			0.04	0.25
Adenocarcinoma	474	31 (6.5)		208	18 (8.7)		
Mucinous adenocarcinoma	18	0 (0)		14	1 (7.1)		
Undifferentiated carcinomas	6	1 (16.7)		4	1 (25.0)		
Unknown	24	2 (8.3)		22	6 (27.3)		
Preoperative radiotherapy			0.52			0.10	0.21
No	233	17 (7.3)		124	17 (13.7)		
Yes	289	17 (5.9)		124	9 (7.3)		

^1^ Pearson’s chi-square test

^2^ Chi-square test for trend

^3^ Likelihood ratio test for gender interaction

Table 3 presents the results of the associations between gender and stage IV in each stratum of these two interacting factors: screening status (screen-detected, symptomatic, or unknown referral route) and diagnostic delay (≤ or > 3 months or unknown diagnostic delay) (Table 3). The unadjusted OR for disseminated disease was 6.9-fold higher in women than in men among screen-detected cases and 3-fold higher in women than in men among cases with unknown screening status, while no significant gender differences were observed in the probability of disseminated disease among those that had presented with symptoms. Among patients with a diagnostic delay > 3 months, disseminated disease was more common in women than in men (unadjusted OR = 6.3; 95% CI, 1.6–24.9), while no significant gender difference was found in patients with delays ≤3 months.

**Table 3:** Odds ratios (women vs men) for stage IV disease by screening status and diagnostic delay

	Stage IV			
	Unadjusted^a^		Adjusted^a**,**b^	
	OR (95% CI)	***p***-value	OR (95%)	***p***-value
Screening				
No	1.23 (0.67–2.16)	0.48	1.04 (0.61–1.77)	0.89
Yes	6.9 (0.88–53.96)	0.06	7.20 (0.93–55.79)	0.05
Unknown	3.05 (2.27–4.11)	< 0.0005	4.26 (3.18–5.72)	< 0.0005
Diagnostic delay				
≤ 3 months	1.07 (0.56–2.08)	0.83	0.95 (0.49–1.85)	0.99
> 3 months	6.25 (1.57–24.89)	0.009	5.05 (1.18–21.58)	0.03
Unknown	3.61 (1.19–10.96)	0.02	3.91 (1.13–13.53)	0.03

^a^We used generalized estimating equations to estimate the women-to-men odds ratios (ORs) in each stratum of the variables with significant multiplicative interactions

^b^Adjusted for age, comorbidity, and preoperative radiotherapy

The women-to-men odds ratios were adjusted for age, comorbidities and preoperative radiotherapy. The histological classification was not included in this analysis as it had very few or no observations in some categories and this lack of information hindered convergence of the estimation algorithm. After adjustment, the associations were maintained but the large confidence intervals indicate a lack of precision in these OR estimates attributable to the small sample size in some strata. Power analyses are reported in supplementary material. (Online Resource, Supplementary Table 1).

### Differences in the use of preoperative radiotherapy

Table 4 shows a gradient of decreasing use of preoperative radiotherapy with age and this gradient is sharper in men, with a significant interaction between age and gender (*p* value of LR test for interaction = 0.015).

**Table 4:** Association of preoperative radiotherapy with various factors in men and women

	Preoperative radiotherapy						***p***-value for gender interaction^**3**^
	Men			Women			
	N	n (%)	P^**1**^	N	n (%)	P^**1**^	
Age			< 0.0005^2^			0.32^2^	0.015
< 65 years	221	140 (63.3)		108	55 (50.9)		
65–80 years	245	132 (53.9)		105	56 (53.3)		
> 80 years	56	17 (30.4)		35	13 (37.1)		
Charlson index			0.05			0.02	0.73
0	258	154 (59.7)		165	91 (55.2)		
≥ 1	264	135 (51.1)		83	33 (39.8)		
Tumor depth			< 0.0005			< 0.0005	0.20
cT1 - cT2	91	18 (19.8)		55	10 (18.2)		
cT3	273	211 (77.3)		120	81 (67.5)		
cT4	28	24 (85.7)		34	23 (67.6)		
Unknown	130	36 (27.7)		39	10 (25.6)		
Distance from anal verge			< 0.0005			0.01	0.95
< 5 cm	100	69 (69.0)		57	34 (59.6)		
5–10 cm	218	141 (64.7)		83	48 (57.8)		
> 10 cm	167	67 (40.1)		90	37 (41.1)		
Unknown	37	12 (32.4)		18	5 (27.8)		
Histological classification			0.20			0.63	0.97
Adenocarcinoma	474	267 (56.3)		208	103 (49.5)		
Mucinous adenocarcinoma	18	8 (44.4)		14	7 (50.0)		
Undifferentiated carcinomas	6	1 (16.7)		4	1 (25.0)		
Unknown	24	13 (54.2)		22	13 (59.1)		

^1^ Pearson chi-square test

^2^ Chi-square test for trend

^3^ Likelihood ratio test for gender interaction

As expected, we observed a greater likelihood of pre-operative radiotherapy with increasing tumor depth, in both men and women, although the gradient appeared somewhat steeper in men. The large proportion of missing values of tumor depth both in men and women attenuated the significance of the gender and tumor depth interaction test (p value of LR test for interaction =0.20). Hence, we tested for this interaction in the stratified GEE analyses since the percentage of patients on preoperative radiotherapy among those with large and very large tumors (CT3 and CT4) suggested that it was more commonly used to reduce tumor size in men than in women (77.5 and 85.7% in men vs 67.5 and 67.6% in women).

Table 5 shows the ORs for receiving preoperative radiotherapy, comparing women and men, as a function of these strata. Among patients under 65 years old, women were less likely to receive preoperative radiotherapy. This association was confirmed after adjusting for comorbidity and distance from anal verge (adjusted OR = 0.6; 95% CI, 0.4–1.0), while in individuals over 65 years of age, no significant differences were found between men and women. Among patients with large tumors (cT3-cT4), the women-to-men adjusted OR for receiving preoperative radiotherapy was 0.5 (95% CI, 0.4–0.7), adjusting for comorbidity and distance from anal verge.

**Table 5:** Odds ratios (women vs men) for preoperative radiotherapy by age and tumor depth

	Preoperative radiotherapy			
	Unadjusted^a^		Adjusted^a**,**b^	
	OR (95%CI)	***p***- value	OR (95%CI)	***p***-value
Age^c^				
< 65 years	0.60 (0.37–0.97)	0.04	0.58 (0.34–0.99)	0.04
≥ 65 years	0.96 (0.69–1.31)	0.78	0.90 (0.64–1.25)	0.52
Tumor depth^c^				
cT1-cT2	1.03 (0.47–2.27)	0.72	1.16 (0.52–2.60)	0.72
cT3-cT4	0.58 (0.41–0.84)	0.003	0.51 (0.36–0.74)	< 0.0005
Unknown	0.86 (0.51–1.44)	0.56	0.83 (0.50–1.37)	0.47

^a^ We used generalized estimating equations to estimate the women-to-men odds ratios (ORs)

^b^Adjusted for Charlson index and distance from anal verge

^c^To increase the statistical power, we recoded age into two categories (< 65 and ≥ 65 years) and tumor depth into three categories (cT1-cT2, cT3-cT4 and Unknown)

For each outcome, we carried out sensitivity analysis using only cases with complete data. Results are presented in supplementary material (Online Resource, Supplementary Tables 2 and 3). Regression coefficients were of similar magnitude but standard errors were larger than in the analysis including the categories for missing information.

## Discussion

Our results indicate that a) women are more likely to have disseminated disease at diagnosis, among patients with cancer detected in screening programs and among patients with a diagnostic delay longer than 3 months; and b) women are less likely to receive preoperative radiotherapy, among patients who are younger than 65 and among patients with large tumors.

### Disseminated disease

Studies on colorectal cancer have described a higher risk of advanced disease in women [1–4, 27], but none have assessed whether these differences are associated with patient or tumor characteristics. In one study, researchers observed a non-significant trend towards diagnosis at a later stage in men [8]. In rectal cancer in particular, three recent studies have contradictory results. Katzenstein et al. [28] and Lydrup et al. [29] reported that tumor stage was similar in men and women while Martling [18] reported slightly earlier stages in women than in men.

In colorectal cancer, the relationship between diagnostic delay and tumor stage is controversial in part due to differences between studies, specifically, differences in definitions of types and length of delays. In the case of rectal cancer, less is known given the lack of studies focused on this site. A recent seven-cohort study on colorectal cancer concluded that longer diagnostic intervals are associated with more advanced stages of the disease [30] and the results of the Spanish cohort indicated, similar to in our cohort, longer diagnostic intervals in women than in men [16].

The gender differences we observed in diagnostic delay are especially striking in patients diagnosed with disseminated disease, suggesting that the disease is suspected and confirmed earlier in men than in women, both in cases referred from screening and those who presented with symptoms. Insufficient physical examination has been cited as a main cause of delay [31], but it is not known whether there are actually differences between men and women in terms of the examinations or tests conducted to reach to a diagnosis.

In our study, among patients with delays of more than 3 months, the rate of disseminated disease was higher in women than in men. Similar results were reported by Korsgaard et al. [32], who suggested that the difference was a reflection of the higher proportion of women among over-70-year-old patients, as in this age group there was a strong association between delay and advanced cancer. In our cohort, however, over-65-year-old patients did not have a higher rate of disseminated disease than younger patients. Our results suggest that this effect may be attributable to men having to wait less time for a diagnosis.

A recent study on trends in rectal cancer 5-year survival rates states that the longer survival of women could be due to genetic, hormonal or environmental factors [33], suggesting that the introduction of screening could explain the decrease in differences in survival rates by gender in recent years if men were more likely to participate in screening programs, but this is the opposite of what has been observed in the current study and studies conducted in similar European populations [34–37]. An alternative explanation is that the performance of screening tests, in particular, the sensitivity of the FOB test [37–40], is poorer in women. We observed that among screen-detected cases, the rate of disseminated disease was higher in women than in men. Such gender differences in the sensitivity of the FOB test seem to be largest in the first rounds of screening [38], which is relevant for our cohort whose recruitment coincided with the start of the screening programs in the catchment populations of the participating hospitals.

Recently, results have been published from a randomized clinical trial demonstrating that, after 15 years of follow-up, screening with sigmoidoscopy reduces mortality among men but not among women [41]. Although in colon cancer there are known tumor anatomical and physiological characteristics that may explain these differences [21], there is no such evidence in the case of rectal cancer. On the other hand, it has recently been suggested that the peak in incidence of rectal cancer is earlier in men than in women, and hence, the age range for screening (50–69 years old) might be too young to identify many of the types of cancer that develop in women [42].

### Preoperative radiotherapy

We found that women under 65 years of age are less likely to receive preoperative radiotherapy than men of the same age, regardless of having the same level of comorbidity and tumor depth. Previous studies have reported less use of preoperative radiotherapy in women [17–20, 28, 29, 43]. Unlike our results, Paulson et al. found that the differences in preoperative radiotherapy between men and women occurred in those older than 80 years of age. For this part of our analysis, however, given sample size limitations, we classified patients as < or ≥ 65 years old and the gender difference was only found in the younger patients.

A second factor related to a lower use of radiotherapy in women was tumor depth: among patients with large tumors at diagnosis, women were less likely to receive preoperative radiotherapy. Among patients with cT4 tumors, women had a higher rate of invasion of neighboring organs [5]. Such invasion complicates the administration of radiotherapy and increases the risk of local complications [44], in particular, rectovaginal fistulas. This might explain a tendency to not use preoperative radiotherapy as an initial treatment in women with stage T4 rectal carcinoma with local and regional invasion of an anterior organ. It is not an absolute contraindication to radiotherapy but it is a controversial issue in clinical practice.

Anatomical differences between the pelvis of men and women and their implications for the difficulty of the surgical technique could be another explanation. In particular, in men, who have a narrower pelvis, it might be more difficult to achieve tumor-free circumferential resection margins, implying a greater need for radiotherapy. The scientific evidence available does not support this thesis, however. According to Jeyarajah et al. [45], a Total Mesorectal Excision score of 3, indicating optimal surgical resection, was significantly more likely in men than in women.

Comorbidity could be a contraindication to adjuvant radiotherapy [46]. We found a higher level of comorbidity to be associated with less use of preoperative radiotherapy, and in line with other authors [17, 47], the levels of comorbidity were higher in men. If an influence of comorbidities were to underlie gender differences, the rate of preoperative radiotherapy would be lower in men, but this is the opposite of what we observed.

### Strengths and limitations

The main strength of this research is its hypothesis of gender as an effect modifier and therefore, the use of a) gender-stratified analyses of associations between outcomes and known determinants, and b) tests of multiplicative interactions between gender and those determinants. Although conceptually gender is the factor that modifies the effects of age and the clinical factors on rates of stage IV at diagnosis and preoperative radiotherapy, we have chosen to present the associations of gender with the two outcomes (ORs for women compared with men) seeking to illustrate that being a woman may result in lower chances of early diagnosis and lower chances of preoperative radiotherapy, all other factors being equal. Furthermore, these analyses have revealed gender differences in rectal cancer diagnosis and treatment that would not have been apparent in a risk factor analysis adjusting for gender as a potential confounder.

Additional strengths of this study are the participation of a large number of public hospitals from different geographical areas in Spain. This implies a great diversity of patients covering a wide spectrum of socioeconomic levels and clinical characteristics. Further, we should note two methodological characteristics that strengthen the internal validity of our findings: a) recruitment and data collection were prospective; and b) data were gathered on numerous clinical characteristics potentially associated with the outcomes, enabling us to address potential confounding factors, as well as investigate in which groups of patients and types of tumors any differences observed occur.

The limitations of this study include:

- Findings are restricted to patients who underwent surgery
- We are not able to distinguish between screen-detected cases from population screening and opportunistic screening in primary care. Nonetheless, although the strength of the association between screening and stage IV might be influenced by the type of screening, we have no reason to believe that it would depend on gender.
- Data were missing on tumor depth for a relatively high percentage of patients (22%). It is likely that these patients with missing data had small tumors, given that their rate of radiotherapy was similar to that in patients with small tumors (and very different from that in patients with large tumors). Further, the use of radiotherapy did not differ significantly between men and women with an unknown tumor size.
- We have conducted multiple comparisons, which increase the probability of reporting spurious associations but the multiple testing has been done according to a recommended analytical strategy for testing gender-linked and gender interactions with potential risk factors as opposed to routinely adjusting for gender or simply reporting gender differences without examining potential effect modification [48].
- In the analyses of disseminated disease, the large confidence intervals of the estimated women vs men odds ratios indicate that these results are based on few observations in some cells. Therefore, results should be confirmed in large cohorts.

## Conclusions

In conclusion, we reported that among patients undergoing surgery for rectal cancer, the risk of having disseminated disease at diagnosis are higher among women when the disease is detected through screening and when there is a diagnostic delay of more than 3 months. Further, among patients under 65 years old or with large tumors, women are less likely to receive preoperative radiotherapy.

These findings open new lines of research on establishing gender-specific cut-offs for FOB tests and investigating whether there are gender differences in the clinical management during the first visit and referrals to specialists and whether there are clinical reasons for not prescribing preoperative radiotherapy in young women and/or women with large tumors. If these gender differences are not clinically justifiable, their elimination might enhance survival.

## Supplementary information

**Additional file 1 Table S1**. Power analysis. **Table S2**. Sensitivity analysis for Stage IV. **Table S3**. Sensitivity analysis for Preoperative radiotherapy.

## Data Availability

Due to a lack of consensus among researchers at all participating centers, raw data is not provided.

## Abbreviations

AJCC: American Joint Committee on Cancer
CI: Confidence Interval
FOB: Fecal occult blood
GEE: Generalized estimating eq.
LR: Likelihood ratio
MRI: Magnetic resonance imaging
OR: Odds Ratio
