# Supplementary material for: Gender differences in stage at diagnosis and preoperative radiotherapy in patients with rectal cancer
Source: BMC Cancer. 2020 Aug 14;20:759. doi: 10.1186/s12885-020-07195-4 (PMC7427942; doi:10.1186/s12885-020-07195-4)
Supplement: Supplementary file 1 — Additional file 1 Table S1. Power analysis. Table S2. Sensitivity analysis for Stage IV. Table S3. Sensitivity analysis for Preoperative radiotherapy. [file 12885_2020_7195_MOESM1_ESM.docx]

**Power analysis**

We have performed power calculations using the free software offered by E. Demidenko to compute power in logistic regression with binary covariates and interactions.

<https://www.dartmouth.edu/~eugened/power-samplesize.php> and explained in the paper: Demidenko E. Sample size determination for logistic regression revisited. *Stat Med*. 2007; 26(18):3385-3397. doi:10.1002/sim.2771.

As expected from the wide confidence intervals in the GEE logistic regression models, power for the gender and exposures interactions is generally low as shown in the table below:

Supplementary Table 1. Power analysis

| OUTCOME | N | Pr(X=1) | Pr(Z=1) | OR(X/Z) | OR(Y/X) | OR(Y/Z) | OR  interaction | PR(Y=1/ X=0,Z=0) | Power |
| --- | --- | --- | --- | --- | --- | --- | --- | --- | --- |
| **Preoperative**  **radiotherapy** |  |  |  |  |  |  |  |  |  |
| Gender*age | 770 | 0.42 | 0.32 | 0.95 | 1.76 | 0.99 | 0.60 | 0.50 | 0.37 |
| Gender*tumor depth | 601 | 0.76 | 0.34 | 0.85 | 14.4 | 0.90 | 0.65 | 0.20 | 0.14 |
| **Stage IV** |  |  |  |  |  |  |  |  |  |
| Gender*  screening | 728 | 0.13 | 0.32 | 0.94 | 0.40 | 1.22 | 5.37 | 0.07 | 0.43 |
| Gender* diagnostic delay | 671 | 0.21 | 0.32 | 1.71 | 0.29 | 1.09 | 5.83 | 0.08 | 0.52 |

We present this power analyses as complementary material and we refer the reader to it in the main text. Since most current epidemiological research uses confidence interval estimation, we have acknowledged the lack of precision of the confidence intervals of the estimation on effects in the limitation section of the discussion.

Supplementary Table 2. Sensitivity analysis for Stage IV

|  | Gender and screening | | | |  | Gender and diagnostic delay | | | |
| --- | --- | --- | --- | --- | --- | --- | --- | --- | --- |
|  | Complete data  N=440 | | Unknown data  N=770 | |  | Complete data  N=440 | | Unknown data  N=770 | |
|  | β | EE | β | EE | =440 | β | EE | β | EE |
| Gender  Women vs Men | 0.04 | 0.33 | 0.09 | 0.27 | Gender  Women vs Men | -0.06 | 0.38 | -0.03 | 0.33 |
| Age  >65 vs <65 | -0.23 | 0.27 | -0.41 | 0.30 | Age  >65 vs<65 | -0.25 | 0.25 | -0.39 | 0.29 |
| Screening:  Unknown vs no  Yes vs no | -  -1.22 | -  1.09 | 0.08  -1.05 | 0.50  0.82 | Screening:  Unknown vs no  Yes vs no | 0.10 | 0.45 | 0.75  -0.13 | 0.29  0.50 |
| Charlson index  >=1 vs 0 | -0.44 | 0.19 | -0.49 | 0.18 | Charlson index  >=1 vs 0 | -0.42 | 0.19 | -0.46 | 0.17 |
| Diagnostic delay (months):  Unknown vs <3 | - | - | -0.20 | 0.53 | Diagnostic delay (months):  Unknown vs < 3 | - | - | -0.76 | 0.63 |
| >3 vs <3 | -0.22 | 0.29 | -0.26 | 0.30 | >3 vs <3 | -1.72 | 0.99 | -1.31 | 0.71 |
| Preoperative radiotherapy  Yes vs no | -1.08 | 0.28 | -0.56 | 0.27 | Preoperative radiotherapy  Yes vs no | -1.06 | 0.31 | -0.52 | 0.29 |
| Gender and screening:  Women *scr unknown  Women * scr yes | -  2.16 | -  1.47 | 1.25  1.79 | 0.44  1.13 | Gender and diagnostic delay:  Women*delay unknown  Women*delay>3 | -  2.31 | -  1.07 | 1.21  1.78 | 0.55  0.81 |
| Constant | -1.42 | 0.29 | -1.78 | 0.31 | Constant | -1.40 | 0.30 | -1.79 | 0.31 |

Supplementary Table 3. Sensitivity analysis for Preoperative radiotherapy

|  | Gender and age | | | |  | Gender and tumor depth | | | |
| --- | --- | --- | --- | --- | --- | --- | --- | --- | --- |
|  | Complete data  N=440 | | Unknown data  N=770 | |  | Complete data  N=440 | | Unknown data  N=770 | |
|  | β | EE | β | EE |  | β | EE | β | EE |
| Gender  Women vs Men | -0.88 | 0.46 | -0.99 | 0.33 | Gender  Women vs Men | -0.22 | 0.36 | -0.08 | 0.32 |
| Age  >65 vs <65 | -0.77 | 0.18 | -0.79 | 0.15 | Age  >65 vs <65 | -0.59 | 0.19 | -0.47 | 0.17 |
| Charlson index  >=1 vs 0 | -0.16 | 0.14 | -0.38 | 0.14 | Charlson index  >=1 vs 0 | -0.14 | 0.14 | -0.39 | 0.13 |
| Tumor depth:  Unknown  cT3-cT4 | -  - 2.50 | -  0.39 | 0.03  2.62 | 0.36  0.30 | Tumor depth:  Unknown  cT3-cT4 | -  2.68 | -  0.39 | 0.13  2.76 | 0.36  0.28 |
| Distance from anal verge (mm): |  |  |  |  | Distance from anal verge (mm): |  |  |  |  |
| Unknown vs <5 | - | - | -1.42 | 0.44 | Unknown vs <5 | - | - | -1.35 | 0.43 |
| >10 vs <5 | -1.55 | 0.38 | -1.40 | 0.30 | >10 vs <5 | -1.52 | 0.38 | -1.34 | 0.31 |
| 5-10 vs <5 | -0.60 | 0.41 | -0.45 | 0.28 | 5-10 vs <5 | -0.57 | 0.41 | -0.40 | 0.29 |
| Gender and age:  Women and >65 | 0.43 | 0.44 | 0.92 | 0.36 | Gender and tumor depth:  Women and depth unknown  Women and cT3/cT4 | -  -0.51 | -  0.31 | -0.25  -0.52 | 0.47  0.34 |
| Constant | 0.41 | 0.52 | 0.22 | 0.44 | Constant | 0.13 | 0.48 | -0.09 | 0.43 |
